# Supplementary figures and images for: Expression of EMP1, EMP2, and EMP3 in breast phyllodes tumors
Source: PLoS One. 2020 Aug 28;15(8):e0238466. doi: 10.1371/journal.pone.0238466 (PMC7454950; doi:10.1371/journal.pone.0238466)

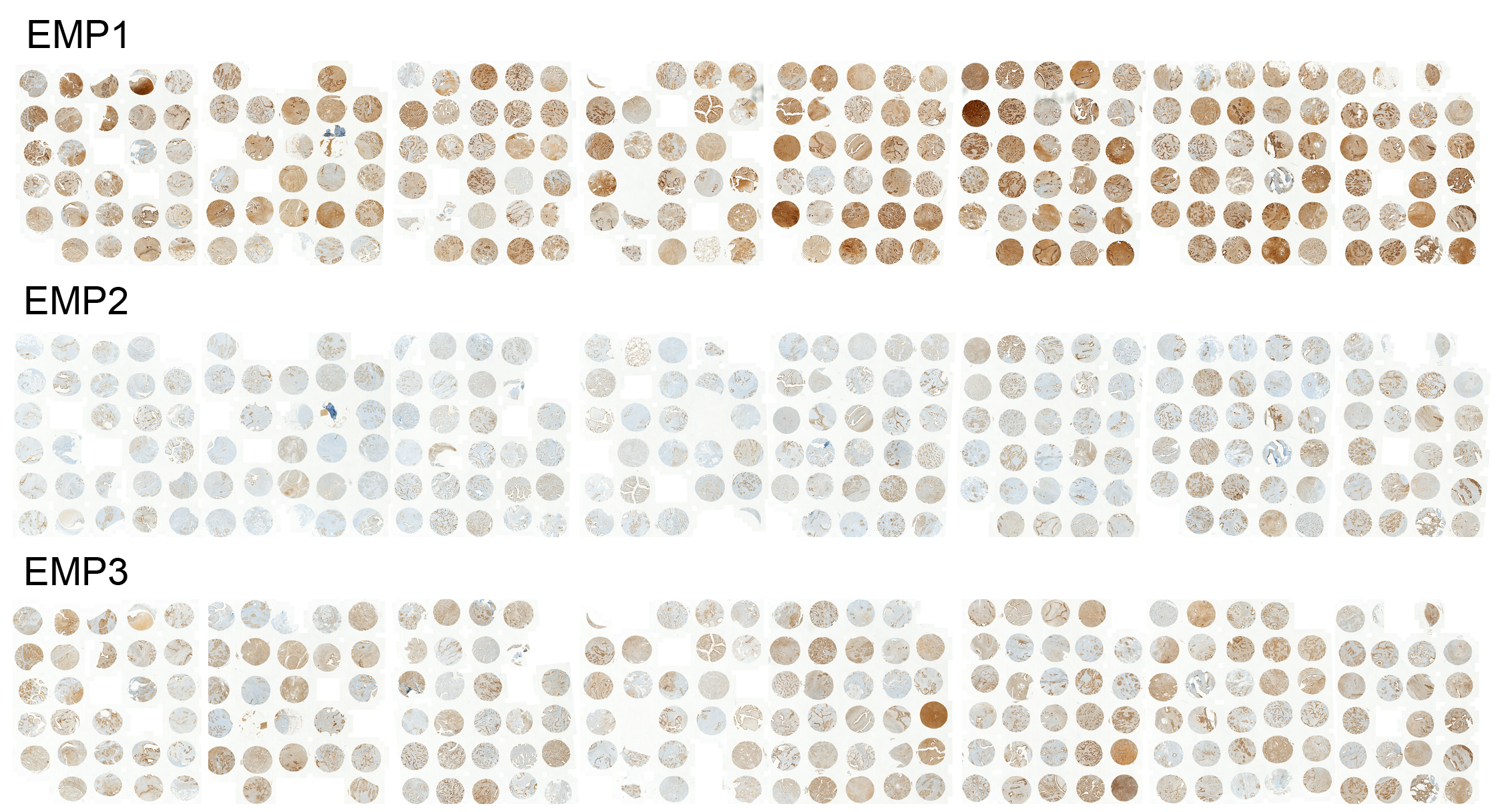

Supplement: S1 Fig — (TIF) [file pone.0238466.s001.tif]

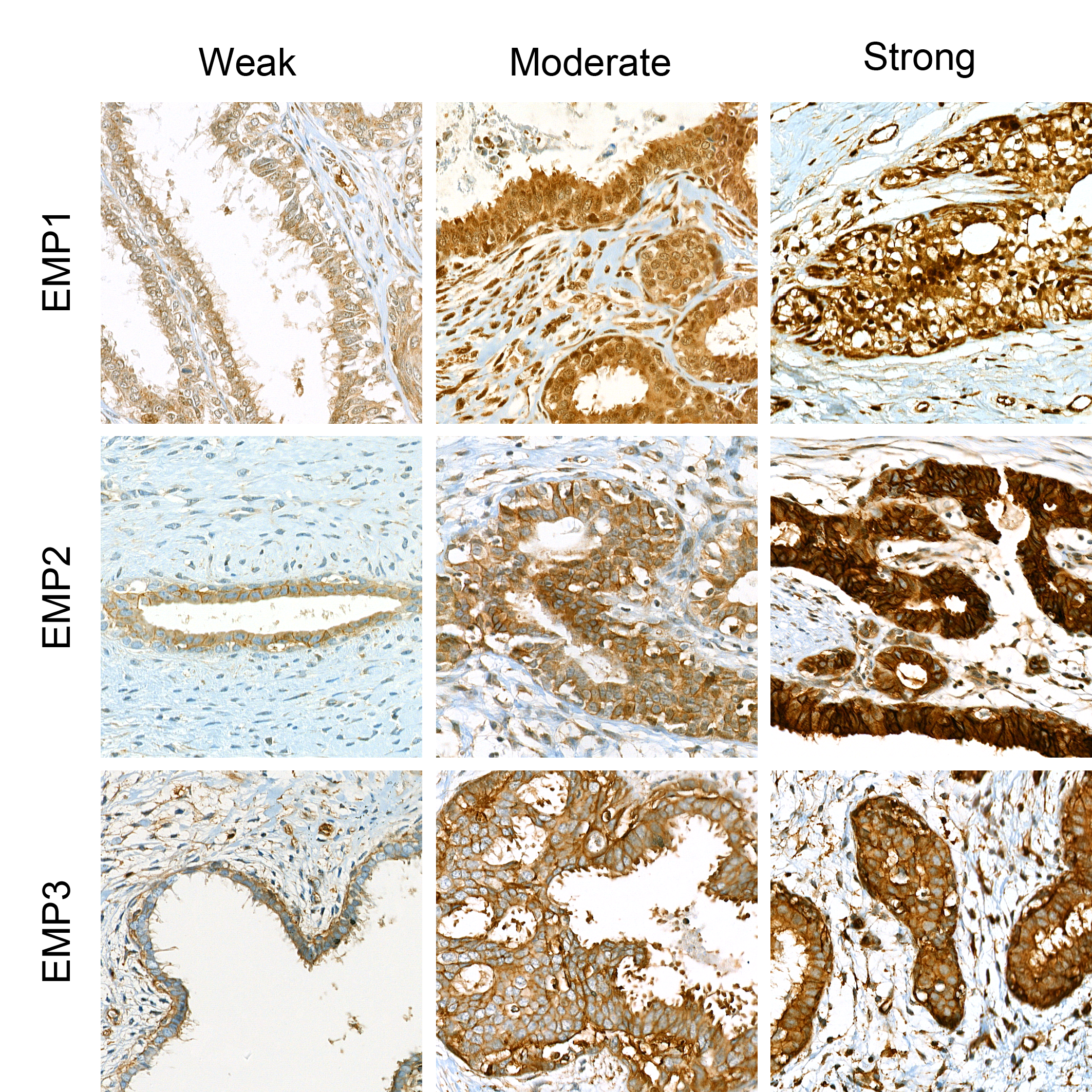

Supplement: S2 Fig — (TIF) [file pone.0238466.s002.tif]
