# Supplementary material for: Expression of EMP1, EMP2, and EMP3 in breast phyllodes tumors
Source: PLoS One. 2020 Aug 28;15(8):e0238466. doi: 10.1371/journal.pone.0238466 (PMC7454950; doi:10.1371/journal.pone.0238466)
Supplement: Attachment — Submitted filename: Reviewer_answer.docx [file pone.0238466.s003.docx]

Reviewer #1:

Thank you for your valuable comments. We revised the manuscript according to your helpful suggestion.

1. Were the pathologists blind to the identity of the patients, their clinic-pathological diagnoses, and survival data?

[Answer] Yes

2. Please provide lower magnification images of all the IHC slides – demonstrating the distribution of EMP staining in the stromal vs epithelial areas.

[Answer] We provide the scan power view of all IHC slides as supplementary figure 1. (page 5, line 2-3)

3. Please provide higher magnification images of all the IHC slides – demonstrating cell membranous localization of the EMP proteins.

[Answer] As there are many EMP positive cases, we provide the representative positive slides with high magnification as supplementary figure 2. (page 5, line 2-3)

4. How was the “proportion of stained cells” calculated? How many areas per tissue were stained for histo-pathological evaluation? How many images were taken for each of the tissue sections? How was “staining intensity” determined? On what basis did the authors use “30%” as the cutoff to determine positivity of EMP staining? Please explain with appropriate references.

[Answer] Proportion of stained cells was evaluated by stained area within the TMA tissue cores. In this study, we used two 5mm tissue cores per each case. Most of stained cases demonstrated homogenous staining pattern.After review of every slides of each case, most representative areas were extracted for TMA construction. Generally, phyllodes tumor smaller than 4-5cm, all tumor content is made into paraffin block and examined with HE slides. Tumors bigger than 6cm, at least one block per centimeter is submitted for diagnosis.

As supplementary figure we submitted, we scanned every TMA slides for taking pictures. In case of high magnification, 9-10 images can be obtained per each core.

Staining intensity is divided by three groups, and defined relatively in each antibody. Generally, in EMP2 and EMP3, weak intensity was recognizable positivity at high magnification (400x). Strong intensity was intense positive that easily recognized under low magnification (12.5x). Moderate intensity was defined between weak and strong intensity. In EMP1, expression intensity tended to be generally strong, most of the intensity was 2 or 3.

We applied the staining scoring system from the previous study (PMID: 19762066). In PTs, EMPs were homogenously stained in most of cases, so in many cases, positivity was determined by staining intensity.

Reviewer #2: Authors investigated the relationship between the expression of EMP1/2/3 and clinicopathological features of phyllodes tumors, and showed EMP expression is reduced in the epithelial region, while it is increase in the stromal region, along with increasing histologic grade.
Thank you for your valuable comments. We revised the manuscript according to your helpful suggestion.

1) Immunohistochemical staining is quite essential for this study, and its procedure must be standard to obtain solid data. Authors did not use any first antibodies for control. They should use the same class of non-specific antibodies to check non-specific staining, otherwise they could not tell any difference correctly in their staining.

[Answer] We already and always used the positive and negative controls when we set up the appropriate dilution of antibody. Accordingly, we revised the method IHC section. (page 4, line 27-28)

2) Histologic grade (mitosis, atypia, cellularity) is generally thought to be the most reliable variable for phyllodes tumors. Although stromal EMP3 is significantly correlated with prognosis in univariate analysis, stromal EMP3 is not significant in multivariate Cox regression analysis, but histologic grade. This data might lead us to the conclusion that EMPs is not necessarily required for the diagnosis of phyllodes tumor.
[Answer] We absolutely agree with your comment. We also found that the clinical impact of this study and the data might be not that significant, But there was no preceding data of EMP and PT, we wanted to investigate the expression of EMPs in PTs, and found that stromal EMPs are increasingly expressed along with histologic grade of PT, and stromal EMP3 could be an prognostic factor. We hope that our result might help other investigators studying EMPs.

1) Authors describe about the tissue microarray in materials and methods, but no data was found using tissue microarray. Why?
[Answer] All the data in result section is the IHC and statistical results using TMA

Reviewer #3: The manuscript entitled ‘Expression of EMP1, EMP2, and EMP3 in breast phyllodes tumors Yoon Jin Cha and Ja Seung Koo is a descriptive paper aimed to evaluate the expression of Epithelial membrane proteins (EMPs) EMP1, EMP2, and EMP3 in breast phyllodes tumors, and to investigate their clinical implications. This manuscript demonstrates using EMP3 expression may serve as an independent prognostic factor in phyllodes tumors. They used 185 samples in this study, with 138 benign, 32 borderline, and 15 malignant phyllodes tumors.

The subject is interesting and significant, the approach is correct. This is a generally well-written and easy to follow. The tables and figures presented in this manuscript are clear.
[Answer] Thank you for your comments.

Reviewer #4: This is a clear, concise report. It will be of value to those interested in the EMP proteins and those investigating the biology of Phyllodes tumors. It is rare to see a report ready for publication on first submission, but this meets all the requirements for publication in PLOS One.
[Answer] Thank you for your comments.
